# Strategies to Fight Stigma toward People with Mental Disorders: Perspectives from Different Stakeholders

**DOI:** 10.1100/2012/516358

**Published:** 2012-10-11

**Authors:** Marc Corbière, Esther Samson, Patrizia Villotti, Jean-François Pelletier

**Affiliations:** ^1^School of Rehabilitation, Université de Sherbrooke, and Centre for Action in Work Disability Prevention and Rehabilitation, 150 Place Charles LeMoyne, Longueuil, QC, Canada J4K 0A8; ^2^Association Québécoise pour la Réadaptation Psychosociale, 2380 Avenue du Mont-Thabor, Bureau 205, Québec, QC, Canada G1J 3W7; ^3^Department of Cognitive Sciences and Education, University of Trento, Matteo del Ben 5, 38068 Rovereto, Italy; ^4^Département de Psychiatrie, Centre de Recherche Fernand-Seguin, Hôpital Louis-H. Lafontaine, Université de Montréal, 7401 Rue Hochelaga, Montréal, QC, Canada H1N 3M5; ^5^Department of Psychiatry, Yale Program for Recovery & Community Health, Yale University School of Medicine, Erector Square, Building One, 319 Peck Street, New Haven, CT 06513, USA

## Abstract

This study aims to provide a more complete and exhaustive perspective on the whole range of potential strategies to fight stigma by considering the perspectives of different stakeholders. Delegates to a Canadian conference were invited to participate in a survey that focused on stigma, from which the responses to the following question were analyzed: tell us briefly what you do to reduce prejudice and stigma toward people with a diagnosis of mental disorder? From 253 participants, 15 categories of strategies to fight stigma were identified from the verbatim (e.g., sharing/encouraging disclosure). These categories fell under six main themes: education, contact, protestation, person centered, working on recovery and social inclusion, and reflexive consciousness. The occurrence of these themes was different among stakeholders (clinical, organizational, and experiential knowledge). For example, people with mental disorders (experiential knowledge) often mentioned contact and person centered strategies, while mental health professionals (clinical knowledge) preferred education and working on recovery and social inclusion strategies. The results from this study highlight the need to pay more attention to the concept of disclosure of mental disorders in the process for de-stigmatization. Future studies are needed to assess the impact of the emerging strategies to fight stigma in the community.

## 1. Introduction

Much has been written about stigma and how it applies to people with severe mental illness [1–3]. Stigma is a complex term defined as a visible or invisible attribute, deeply discrediting, that disqualifies its bearer from full social acceptance, often resulting in several forms of discrimination [4]. Today, stigma is described as “a severe social disapproval due to believed or actual individual characteristics, beliefs or behaviors that are against norms, be they economic, political, cultural or social” [5, p. 10]. It is characterized by a lack of knowledge about mental health, fear, prejudgment, and discrimination. In its most advanced forms, stigma leads to exclusion of the person from several spheres of social functioning and it causes feelings of guilt, shame, inferiority, and a wish for concealment [6].

Stigma toward people with mental disorders is a complex issue with the capacity to affect all facets of a person's life, such as the opportunity to find housing and employment, enter higher education, obtain insurance, and get fair treatment in the criminal justice or child welfare systems [7, 8]. Thus, stigma robs people with mental illness of particularly important life opportunities vital to achieving life goals, obtaining competitive employment, and living independently in a safe and comfortable home [9].

Stigmatization toward people with mental disorders stems from different stakeholders in the community and can be expressed differently, considering these perspectives, sometimes resulting in self-stigmatization. Evans and Repper [10] reported that the general tendency for employers and mental health professionals is to underestimate the capacities and skills of people with mental illness: these behaviors, to a certain extent, can be experienced as discriminating. Lack of interest in the person's background and needs and exclusion of relatives from treatment planning have also been mentioned as professionals' stigmatizing attitudes toward people with mental illness [11, 12]. It has also been argued that mental health professionals can sometimes hold the same public stigmatizing attitudes toward mentally ill individuals as well as very pessimistic views of their chances of recovery [8]. Stigmatizing attitudes have also been observed among students from many segments of medical and psychological services [13]. An additional issue is that some people with mental illness endorse stigmatizing attitudes about psychiatric disability, starting to believe that he/she deserves to be treated in such a way. The internalized stigma affects the individual's self-perception and can potentially impact success or failure in life opportunities, such as employment. This serves to reinforce the negative stereotypes and social exclusion associated with severe mental illnesses [14]. Thus, self-stigma leads people with mental illness and their families to adopt attitudes of self-loathing and self-blame, resulting in a sense of helplessness and hopelessness [8].

More than 40 negative consequences of stigma have been reported in the literature [15, 16]. While the damaging impact of stigma is mainly confined to the stigmatized individual, public stigma also impacts their families and close friends, who can experience high levels of shame and embarrassment [17]. This is what has been called the “courtesy of stigma”, meaning the result of being related to a person with a stigma [4, 11, 18]. In general, everyone who comes into close contact with the mentally ill, such as mental health support groups and even mental health professionals [4, 13, 19, 20], suffers from their own type of public stigma. For example, a psychiatrist's authority has been considered inferior to other medical experts, so patients often ignore their advice and, therefore, they frequently appear ineffective [21].

In sum, stigma can severely and negatively impact mentally ill individuals, their families, and service providers in a number of ways. Due to stigma's devastating effects, studies worldwide have recently aimed to raise awareness and understanding about the most effective strategies to combat stigma and discrimination. Little is known about how to combat stigmatizing attitudes toward people with mental illness and the ingredients for successful antistigma activities [18, 22]. The literature identifies three general approaches for countering stigmatizing attitudes and discriminating behavior associated with mental illness. These are education, contact, and protest [23, 24]. Although each of these stigma-reducing approaches has some degree of validity on the surface, they are not uniformly effective [25].

The first strategy to fight stigma originates from the belief that stigma is related to poor factual knowledge about mental illness and seeks to inform the general public and health professionals by replacing inaccurate stereotypes and false assumptions of mental illness with facts and accurate conceptions about the illness [24, 26]. The limitations of this kind of intervention are that many stereotypes are resilient to change [27], and it has been argued that education modifies literacy and, sometimes, attitudes, but rarely behavior [18].

The second strategy aims to change negative attitudes toward the mentally ill through direct interactions with affected persons. Direct and face-to-face interactions are examples of contact interventions [28]. Contact appears to be the most promising strategy for reducing stigma [27], especially when contact is one-on-one: when people are seen as having equal status and when people are working together in a cooperative rather than competitive manner [29–31]. However, reducing stigma through contact is time-consuming and may not be cost efficient [32]. Also, the efficacy of this strategy seems to depend on the context and the nature of the contact.

The third strategy works on conveying messages to report and to believe reported negative and inaccurate representations of mental illness. Advocacy activities, educational support groups, and patient empowerment groups are examples of interventions within the protest strategy. This kind of strategy is usually effective in diminishing negative attitudes about mental illness but it fails to promote more positive attitudes supported by facts. Also, a rebound effect may occur and can be observed in the stigmatizing beliefs of the public [24, 27], meaning that protest does not necessarily change people's prejudice about mental illness.

The challenge of combating stigma is still prominent in the mental health field and much more needs are to be done. The fight against stigma is a complex endeavor, with multifaceted implications, and must be examined from multiple perspectives (e.g., mentally ill individuals, their families, and healthcare professionals) to increase knowledge and experience about the best strategies for antistigma campaigns. Until now, few studies focusing on the perspective of those having mental illness, relatives or mental health practitioners, have been published and there is a paucity of research using everyday life settings for examining strategies to fight stigma. Most efforts have focused on directly improving community attitudes even though it seems relevant that antistigma programs would also address patients and their relatives. Studies conducted in this manner reported few suggestions, which were mainly concerned with improving information on mental health issues for the public [12, 19].

The main objective of this study is to provide an exhaustive perspective on the whole range of strategies to fight stigma used by different stakeholders, such as mentally ill individuals, their families, mental health professionals, and other people working in mental health organizations. The intent is to focus on everyday and practical strategies that can, ideally, be applied across various settings, such as health, community, workplace, and school. More particularly, specific objectives aim to (1) produce emerging strategies to fight stigma that consider the perspectives of different stakeholders groups; (2) compare the occurrence of different types of strategies to fight stigma according to different types of knowledge: organizational (i.e., directors, managers, or coordinators working in the field of mental health), clinical (i.e., mental health professionals and/or clinicians), and experiential knowledge (i.e., users of mental health services).

## 2. Methodology

### 2.1. Procedure

In November 2010, the Quebec Association for Psychosocial Rehabilitation (AQRP) held its fifteenth conference entitled: “Overcoming Stigma, a Collective Challenge!”. This event brought together over 800 delegates from the public and community sectors of mental health (people who use mental health services, professionals, researchers, managers, etc.). The main objective of this event was to promote collective reflection on the consequences of stigmatization or destigmatization toward people with a mental disorder. As part of this conference, another objective was to enable understanding and familiarization of approaches, actions, resources, and strategies to overcome stigma and promote destigmatization.

At the beginning of the event, the conference delegates were invited to participate in a survey that focused on stigma (see below for a description). The survey invitation was delivered by direct contact: located in strategic areas and at appropriate times (mainly on the first day, during registration and breaks); volunteers invited delegates to a room reserved for data collection. The survey could be answered online (online survey created with SurveyMonkey) or on paper. The survey was approved by the ethics committee of the Université de Sherbrooke.

### 2.2. Survey Description

The questionnaire was developed by a subgroup of the scientific committee of the fifteenth conference of the AQRP. The survey was composed, in part, of a series of questions (Likert scale) from an existing questionnaire. (The questionnaire is found in an ongoing project: Study of Factors Influencing Return-to-Work of People with Depression in (2009) by M. Corbière, M. J. Durand, M. F. Coutu, L. St-Arnaud, T. Lecomte. The project is funded by CIHR and IRSST.) Other questions (open) were developed by the committee. Three people with a mental disorder tested the questionnaire before data collection to ensure the clarity of the questions.

The results presented in this paper relate to one open-ended question of the survey, that is, the strategies used by respondents to reduce prejudice and stigma toward people with a diagnosis of mental disorder. The question was worded as follows: tell us briefly what you do to reduce prejudice and stigma toward people with a diagnosis of mental disorder.

### 2.3. Participants

Every conference delegate was eligible to participate in the study. Of the 801 delegates, 315 agreed to answer the questionnaire. (Please note that it is not possible to establish a precise response rate because the conference was held on 3 days (November 8-9-10, 2010) and data collection was done at the beginning of the conference, at which time not all delegates were present.) A total of 277 people answered the question specific to this paper: 121 (44%) were clinicians/professionals and 74 (27%) were users of mental health services. The other types of respondents each comprised 10% or less of the participants: managers (10%), coordinators (8%), professors-researchers/research professionals/teachers (3%), parents/friends (3%), students (1%), and other (4%). Our sample included 183 women (71%). Considering the whole sample, 168 respondents (62%) held a university degree, nearly a quarter (24%) had a college degree, and (14%) had a high school diploma or less. The age groups of the respondents included 154 (56%) people between 35 and 54, with the remaining respondents either under 35 (21%) or over 54 (23%).

### 2.4. Data Analyses

The authors (M. Corbière and E. Samson) read the verbatim to get a general idea of the strategies mentioned by the respondents. After independently identifying categories of the strategies observed in the verbatim, the authors, together, established a final list of 15 categories of strategies, which excluded nonrelevant or incomprehensible references. The process of establishing categories reduced the number of selected respondents to 253. Because the verbatim was generally simple and straightforward, the strategies mentioned by the respondents were easy to conceptualize. From this common list, the authors conducted categorization of all verbatim independently, then compared the category or categories(s) awarded by each of them to each transcript. The concordance rate for the categories between the authors was high, approximately 92%. Disagreements between the two authors were mainly about categories of strategies that were close in content. For example, “I refuse to speak against people that have a mental disorder” was categorized as “reframing words” by one author and as “defending rights” by the other. These differences were discussed until a consensus was reached.

**Figure 1:**
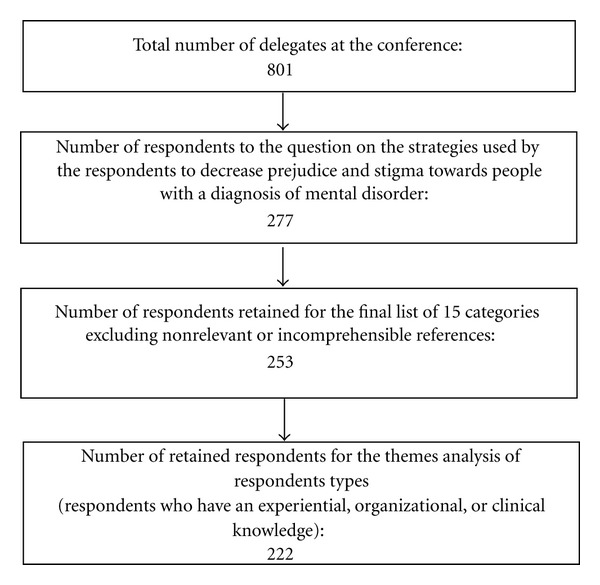
Number of respondents according to the different steps of the analysis.

To distinguish whether the strategies used to fight social stigma differed between the respondents, the 15 categories of strategies obtained were grouped into six major themes inspired by the literature on the subject [23]. Three groups of respondents were also created, according to their type of knowledge: clinical, experiential, and organizational. Respondents matching the clinical knowledge profile were professionals and/or clinicians (*n* = 115) working with people with a mental disorder. People with an experiential knowledge profile were those who, in the survey, identified themselves as users of mental health services (*n* = 61); finally, those who worked in the field of mental health as directors/managers (*n* = 24) or coordinators (*n* = 22) fit the organizational knowledge profile. Since the percentage of other respondents (e.g., professors-researchers/research professionals/teachers (*n* = 8), parents/friends (*n* = 7), students (*n* = 2) was very low (lower than 4% each category), they were not considered for analyses. In the end, respondents with a profile that corresponded to the three targeted types of knowledge (*n* = 222) were included in comparison analyses.  Figure 1 presents the number of retained respondents according to the different steps of the analysis.

## 3. Results

### 3.1. Part 1

As mentioned above, afirstanalysis of the resultsobtained from the253 respondentswhoseentries to the question “Tell us briefly what you do to reduce prejudice and stigma toward people with a diagnosis of mental disorder” produced several strategies that were grouped into 15 categories.  Table 1 shows the occurrence of each category. (The occurrence of each category is the result of dividing the number of respondents who gave a response associated with the category by the total number of respondents to the 15 categories (253). A given respondent may cite more than one strategy.)

We observed that the strategies used addressed not only the general population, but also the people directly concerned by the illness (AQRP conference delegates), which explains the particular nature of strategies used to fight stigma. Indeed, the strategies mentioned in this study can be viewed from three different perspectives depending on who the action targeted: the general population (e.g., educating/teaching); the person with a diagnosis (e.g., working on social inclusion); or the respondent himself (e.g., doing introspective work).

The most commonly mentioned type of strategy, *Educating/teaching* (42%), is a strategy directed at the general population. It aims to inform people and to correct misconceptions with facts.

**I downplay what presents itself and make people aware of what is mental health. I take this opportunity to explain what it can mean to the person, demystify what is happening and bring the person to understand what is happening. (Coordinator)**

A third of the respondents (32%) also mentioned strategies calling for normalizing. In most cases, normalizing was observed as a strategy directed at the person with a diagnosis. This meant treating or considering this individual the same as any other person, looking at that person the same way as anyone else, without any distinction related to the diagnosis, nor to a specific behaviour or opinion.

**I act normal, I treat them like whole people and I ignore the illness. (Clinician/professional)**

**Having the same attitude, the same look as I have for others. (Clinician/professional)**

In some cases, *Normalizing *was a strategy directed at the general population. People with a mental disorder were then presented to others as people who have the right to be different people. The notion of demystification was also present in this category.

**I often tell peoplethat mental healthis very much likephysical health[...]for me, treating mental health isthe same as treating physical health,a good doctorwith agood treatment, goodwill to want torecover.You canlive in the communitylike everyone else. (User of mental health services)**

These two strategies, *Educating/teaching* and *Normalizing* were the two main strategies mentioned by all respondents. Two other strategies also emerged: *Working on Recovery* (19%) and *Working on social inclusion* (15%)

**I work as an occupational therapist in mental health among people with a diagnosis of mental disorder. I accompany them, help them realize their life plan based on their strengths and own difficulties. (Clinician/professional)**

**As a specialized mental health educator, it is part of my work to reduce prejudice by doing the most possible integration into the community with people with a mental health problem. (Clinician/professional)**

These two strategies were directed at the person with a diagnosis. To work on recovery involved supporting, assisting, and encouraging the person. It was about believing in the person, building on his or her strengths and possibilities, rather than taking charge. The respondents identified these attitudes and behaviors as ways to reduce prejudice and stigma. The second strategy, *Working on social inclusion*, referred to promoting the integration of the person with a mental disorder in the community, for example, in terms of social activities, education, or employment.

*Sharing/encouraging* disclosure were strategies directed at the general population and were used by about one in ten respondents (9%). This meant, for people with a diagnosis, disclosing their condition in appropriate circumstances or more formally sharing their story with the public. For people working with individuals with a diagnosis, it meant allowing them to share their story.

**I tell my story. (User of mental health services)**

**I disclose my illness to my employers despite prejudices. (User of mental health services)**

**Have people share their story in front of certain audiences. (Coordinator)**

Other categories of strategies are listed in Table 1, three of which will be discussed here: *listening/caring* (11%), *accepting/respecting* (8%), and *meeting/coming* close to (3%). These three categories of strategies were directed at the person with a diagnosis. It was interesting that the more the category of strategies involved a significant degree of proximity between the respondent and the person with a diagnosis, the less it was mentioned. Thus, while 11% of respondents mentioned that they listen, welcome, and take an interest in the person, and that 8% say they respect, accept, and do not judge the person, only 3% mention meeting, coming close to the person, and making the person a friend, a spouse.

In addition, two other strategies deserve our attention despite their low incidence: *doing introspective work* (6%) and *being natural* (2%) were two self-directed categories of strategies. *Doing introspective work* involved focusing on personal prejudices, ignorance, and working to reduce self-stigmatization.

**I learn to better understand their reality, to correct my perceptions. (Clinician/professional)**

**I don't stigmatise myself. (User of mental health services)**

Conversely, the person with a mental disorder may also choose simply to act naturally (*Being natural*), without publicly disclosing his or her diagnosis. At first glance, this strategy may seem to contradict the notion of sharing. However, the person living with a diagnosis who is acting, day-to-day, like everyone else, without reference to diagnosis, symptoms, or treatments, for example, normalizes mental illness for those she/he meets. For example, the following is the verbatim of a respondent who identified herself as a user of mental health services:

**I live with a diagnosis of mental disorder with being myself. Therefore I become a living model, and since it is not written on my forehead, my mental disorder is part of me and I do not think it is a nuisance. I do not feel compelled to tell everyone. To counter the prejudice and stigma, I chose to act like a person without distinction. (User of mental health services)**

### 3.2. Part II

To determine if the nature of the strategies used varied among types of respondents, the authors placed the respondents into three groups according to their type of knowledge: clinical (*n* = 115), experiential (*n* = 61), and organizational (*n* = 46), as defined above. In addition, the 15 categories of strategies identified initially were grouped under six main themes: education, contact, protestation, person centered, working on recovery and social inclusion, and Reflective consciousness, as presented in Table 2.

The themes *Education, Contact,* and *Protestation* were inspired by the literature on the subject and they refer to three proven strategies to fight stigma [23, 33]. The theme *Education* aims to reduce stigma by providing accurate information about mental disorders. The strategies within this theme rest on the assumption that a better understanding of mental disorders will cause people to reduce their prejudices and act in a nondiscriminatory manner toward individuals who live or has lived with a mental disorder [34]. The theme *Contact* promotes positive interpersonal interactions between a person who has or have lived with a mental disorder (who disclosed his/her condition) and a member of the public; living libraries are an example of the application of this strategy [35, 36]. (Organized in a public place, the living libraries allow the public to “borrow” time (30 min) from a person who has or had a mental disorder and have a conversation with her.) The theme *Protestation* addresses inappropriate or negative representations of mental illness used by the public or media by denouncing them. Some authors include the strategies used by organizations for the defense of rights in the strategies of protestation [37], while others see them as a separate strategy [22].

In the context of this study, the theme *Education* includes strategies from the following categories: *Educating/teaching, *giving successful examples, acting on an organizational level and paying attention to language. *Contact* refers to the strategies that correspond to the categories *Sharing/encouraging disclosure* and *Meeting/coming close to*. *Protestation* is the theme for *defending rights* and *reframing words*.

**Figure 2:**
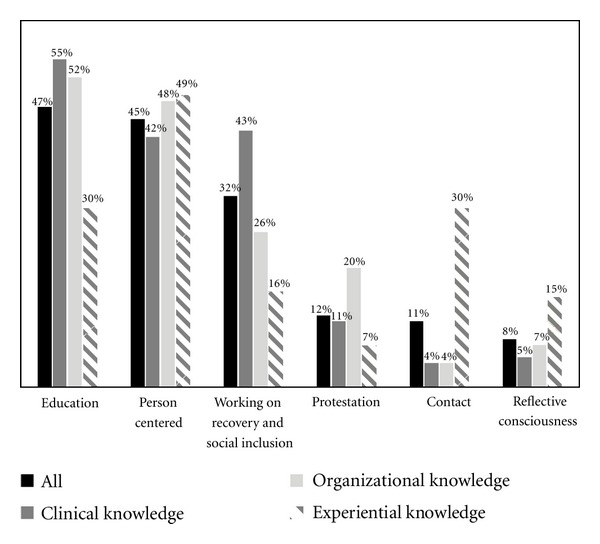
Types of strategies (themes) according to the respondents' type of knowledge.

In addition to these three themes of strategies directly inspired by the scientific literature, this study, which was aimed at people related to the mental health field, has identified three additional major themes of strategies: person centered, working on recovery and social inclusion and reflective consciousness. The theme *Person centered* implies treating the person with a mental disorder diagnosis as any other person (as seen above), but also accepting, respecting, listening to, and caring for the person. This last theme means to act without discrimination against a person with a mental disorder, to welcome that person like anyone else; it is to have speech and values that place the individual as a whole person, beyond diagnosis. The theme *Working on recovery and social inclusion* implies the idea of supporting and encouraging the person, believing in him/her, building on his/her strengths and possibilities, and fostering his/her integration into the community. The theme of *Reflexive consciousness* refers to *Doing introspective work* and *Being natural*. The strategies related to this theme imply a return to oneself. Based on these six broad themes of strategies to fight stigma, Figure 2 highlights the percentages of the three groups of people according to their type of knowledge: experiential, organizational, and clinical.

Figure 2 shows some important distinctions in the strategies used by the three groups of respondents in the study. People with experiential knowledge were easily distinguished from the other two groups. First, they were less likely to have mentioned a strategy related to the theme *Education* (30% versus 55% and 52% for those with clinical knowledge and organizational knowledge, resp.). Second, they were more likely to have mentioned strategies related to the theme of *Contact* (30% versus 4% for those with a clinical profile or organizational). People with *clinical* knowledge mentioned strategies like *Working on recovery and social inclusion* more often than those with *organizational* knowledge and *experiential* knowledge (43% versus 26% and 16%, resp.). In addition, the theme of *Person centered* was a strategy widely used by the three groups studied: nearly half (between 42% and 49%) mentioned it. Strategies grouped under the themes of *Protestation *and *Reflexive consciousness* were less cited by these three groups (between 5% and 20%). Figure 2 shows that, compared to the other two groups, the experiential knowledge group tended to mention more strategies in connection with the theme *Reflexive consciousness* (15% versus 5% and 7%).

## 4. Discussion

The objective of this study was to describe the strategies used by different stakeholders to fight social stigma toward people with a mental disorder. This study is interesting on two levels. First, it was specifically aimed at people connected to mental disorders (e.g., people with a mental disorder, mental health professionals). Second, the strategies identified were, with few exceptions, strategies used individually and spontaneously in everyday life, while studies from the specialized literature almost always report strategies used in structured programs or initiatives [38–41].

From the Canadian conference delegates who answered to the question *Tell us briefly what you do to reduce prejudice and stigma toward people with a diagnosis of mental disorder*, the study identified 15 categories of strategies to fight stigma (e.g., *Reframing words, Working on recovery*). These results underline the creativity of diverse groups of people implementing various strategies to fight social stigma on a daily basis. To our knowledge, few studies have provided these types of results, and this illustrates the importance of consulting different stakeholders to capture the richness and range of opportunities. Indeed, we identified particular categories of strategies rarely cited, such as those relating to *Being natural* or *Doing introspective work,* which display the importance of introspective work for the individual in fighting stigma.

To compare the different stakeholders based on their knowledge—that is, *experiential *(users of mental health services), *organizational* (directors, managers, or coordinators working in the field of mental health), and *clinical* (mental health professionals and/or clinicians)—the 15 previous categories were grouped into six broad themes: *Education, Protestation, Contact, Person centered, Working on recovery and social inclusion,* and *Reflexive consciousness*. As noted in the introduction, the first three themes, *Education*, *Protestation,* and *Contact*, were inspired by the literature on the subject; they refer to three widely recognized strategies to fight stigma [22, 33]. *Education* strategies are very popular because they are readily available to the public, in the case of campaigns, or transferred from one organization to another, in the case of more or less long-term education and awareness programs [23, 39]. *Protestation* strategies aim to reduce stigma by denouncing inaccurate messages. The concepts of testimony and disclosure are also very present in the literature on mental illness stigma. We refer here to the strategy of *Contact* that encourages interactions between a person with a mental disorder and a member of the general public [27].

The other three major themes that emerged from this study were the strategies of *Person centered, Working on recovery and social inclusion,* and *Reflexive consciousness*. Strategies within the theme *Person centered*, used in large proportion (50%) by all types of respondents, are similar to an approach described by Davidson [42] which states that one way to fight stigma toward people living with a mental disorder is to modify the elements that contribute to identifying them, through the eyes of the general population, as people with a mental disorder. The strategies grouped under the general theme *Working on recovery and social inclusion* refer to the psychosocial movement of rehabilitation in psychiatry linked with the (re)construction of personal identity [42, 43]. The concept of recovery is based on the hopefulness of a better life, both inside and outside the network of mental health. These new avenues for the recovery of the individual share the concerns and values of current psychiatric rehabilitation [44]. *Reflective consciousness* strategies are reminiscent of the principles from the reflective approach (or reflection). *Reflective consciousness* is generally defined as a process by which a person reflects and attempts to restructure one's experience and/or knowledge and, consequently, to deal with attitudes and behaviours as objects of observation—in this case toward the social stigma against people with a mental disorder. 

The three major themes of the literature-inspired strategies to fight stigma can have both positive and negative results. While *Education* can help to change attitudes, the magnitude and duration of these changes may be limited [23]. Stuart [34] also emphasized that it is very likely that massive public education campaigns may be weak or ineffective as a contributor to changes in attitudes and behaviors. It has been generally observed that although *Protestation* strategies may remove certain media messages detrimental to people with a mental disorder at the individual level, they can also cause a “rebound” effect, reinforcing the behaviour we wish to eliminate. People targeted by the protest may have the opposite of the intended reaction because nobody likes to be told what to do, say, or think [23, 45]. It has been observed that when the general population interacts with a person with a mental disorder as part of an antistigma program, *Contact* strategy may result in significant improvements in attitude. Moreover, changes in attitudes resulting from these contacts are maintained through time and are related to changes in behavior [2]. The results obtained in this study show that a much higher proportion of respondents with experiential knowledge (those who live or have lived with a mental disorder) use *Contact* strategies to fight against stigma than do respondents with clinical or organizational knowledge. Those with clinical or organizational knowledge can be distinguished from those with *experiential knowledge* by the former's use of *Education* strategies. Note that the *Contact strategy* implicitly involves the concept of disclosure. According to Corrigan and O'Shaughnessy [23], a way to massively increase the power of contact is to encourage people with mental disabilities to disclose their experience. Those who actually do disclose their experience can contribute significantly to fighting stigma. However, some disadvantages can be associated with disclosure: social avoidance by people who know and discrimination in employment or housing. Thus, it is hardly surprising that a few respondents with *clinical* or *organizational knowledge* have cited encouraging disclosure as a strategy they use to reduce stigma, given the disadvantages their clients could encounter.

Strategies within the theme *Person centered* create physical or relational environments that enable a person to begin the process of reconstruction of citizenship identity through the development of interconnectedness capacities, therefore gradually becoming an integral part of society [46]. This theme is intimately linked to the theme of *Working on recovery and social inclusion*. Thus, it is no surprise that people with *clinical knowledge* make significant use of both *Person centered* and *Working on recovery and social inclusion* in similar proportions (42-43%) since their work is based on social inclusion and recovery of people with mental disorders, as well as to help them attain full citizenship [47]. Conversely, adopting *Doing introspective work* or *Being natural* strategies, from the general theme of *Reflexive consciousness,* allows better development of self-knowledge and, therefore, helps the individual to adopt behaviours and attitudes that are closer to social inclusion or even destigmatization.

Based on the results of this study, the theme *Contact* has emerged as an important strategy for people with a mental disorder diagnosis. Given that the literature emphasizes its efficiency to combat social stigma, it is important to better understand the phenomenon of disclosure because it is a key element in the fight against stigma. Several interesting questions arise: what is the experience of people who have disclosed their mental condition? What advantages and disadvantages have they experienced? what are the reasons why some people are not afraid to disclose their condition? Are there beneficial conditions that allow or promote disclosure (e.g., security acquired in housing, employment, social network)? Some of these questions are already answered in the literature. For example, some studies show that disclosure in the workplace has the advantage of ridding the individual of the stress inherent in hiding one's mental disorder, among others, and creates the possibility of requesting work accommodations [48, 49]. Disadvantages can include being at risk of being treated differently and reducing professional opportunities [49]. During the development of programs to fight stigma using a *Contact *strategy, it is important to be well informed about and to consider the factors surrounding disclosure in order to fully support people who decide to disclose their mental disorder. This knowledge is also important for staff working with people with a mental disorder to help them better support those who voluntarily wish to disclose their mental disorder. Currently, the stigma of mental illness is a major barrier to disclosure: according to the Quebec Ministry of Health and Social Services, 42% of people struggling with a mental health problem have not told their family for fear of being judged [50]. Moreover, according to a Canadian Medical Association report [51], only half of Canadians would tell their friends or colleagues if a family member was diagnosed with mental illness (50%), compared to a wide majority that would speak of a cancer diagnosis (72%) or diabetes (68%) in the family. Disclosure (or testimony) and stigma are highly correlated: the more people with mental disorders willingly talk about their condition, the less mental illness will be stigmatized and the more people with a mental disorder will be inclined to disclose their condition.

Some of the published literature notes the importance of working with health professionals to reduce the stigma faced by people with a mental disorder when they receive services [52]. The strategies adopted here by people with *clinical knowledge* can certainly serve as clues to guide these professionals in their contacts with people with a mental disorder. This refers to *Person centered *and *Working on recovery and social inclusion*. By paying attention to the whole person, beyond the diagnosis and symptoms, as implied by a recovery-based approach, a health care professional could avoid falling into the trap of diagnostic overshadowing, which has adverse consequences for people with a mental disorder. Thornicroft [35] explains that because of their diagnosis, people with mental illness are less likely to receive adequate health care from the health professionals than people who do not have this type of disease: examinations are less thorough, treatments are less complete, and the service offer for these people is diminished.

This study has some limitations. First, it identifies strategies to fight stigma used by various groups of people connected to mental disorders but it does not assess the effectiveness of these strategies. If the strategies of *Education, Protestation, *and* Contact* are well documented in relation to their effectiveness in fighting stigma, those that emerged in this study would benefit from being evaluated, including strategies of *Person centered *and *Working on recovery and social inclusion*, as well as *Reflexive consciousness.*

It is also important to note that this study used a sample of individuals concerned with the subject, who, by their presence at the conference, were immersed in an environment where stigma against people with a mental disorder was clearly denounced (recall the title of the symposium: Overcoming Stigma, a Collective Challenge!). This limit may also be an asset: highly aware of and sensitive to the topic, respondents were probably better able to identify the strategies they personally use to combat the social stigma against those with a diagnosis of mental disorder.

In conclusion, this study aimed to provide a thorough and exhaustive perspective on the whole range of strategies to fight stigma used by different stakeholders. Several categories of strategies emerged from the verbatim, which were organized into six main themes: *Education*, *Contact*, *Protestation*, *Person centered*, *Working on recovery and social inclusion*, and *Reflexive consciousness*. Some strategies were used more often than others by specific stakeholders. Furthermore, the notion of disclosure of mental disorders emerged as a key factor for fighting stigma. Future studies will allow us to assess the impact of these strategies on various settings, such as health, community, workplace, and school.

## Figures and Tables

**Table 1:** Categories of strategies used to reduce prejudice and stigma.

Categories	All	
	(*n* = 253)	
	%	Rank
Educating/teaching		
I try to make people around me aware of prejudice whenever I get the chance by explaining what mental health problems are. (Clinician/professional)	42%	1

Normalizing		
I think that people who receive a diagnosis are like everyone else and they shouldn't be treated differently. (Clinician/professional)	32%	2

Working on recovery		
I help them to keep faith […], I work with their strengths and their potentialities. I think with them, rather than taking charge of them or trying to save them. (Coordinator)	19%	3

Working on social inclusion		
I work with mental health clients, I help them to “mingle” in society through various activities. (Clinician/professional)	15%	4

Listening/caring		
…listening to them, welcoming them. (User of mental health services) I place importance on what the person with the diagnosis thinks and expresses. (Coordinator)	11%	5

Sharing/encouraging disclosure		
I share my story of mental illness. (User of mental health services) Encouraging and supporting people with mental illness to disclose and share their experiences. (Director/manager)	9%	6

Accepting/respecting		
I try not to judge these people. (User of mental health services) Respecting their point of view, opinions on their needs and services received. (Clinician/professional)	8%	7

Reframing words		
I insist that they not be called fools during meetings with others. (Clinician/professional)	7%	8

Giving successful examples		
This can be done by showing specific examples of people who have come out of the hospital and were able to live a normal life, like anyone else. (Clinician/professional)	7%	9

Doing introspective work		
You have to be willing to address these issues, to confront yourself, with respect to people with disabilities, to let go of ideas or imagination linked to ignorance. (Clinician/professional)	6%	10

Meeting/coming close to		
I am close to people with mental health problems and these people are my friends. (Parent/friend)	3%	11

Defending rights		
I campaign for the defence of mental health rights. (User of mental health services)	3%	12

Acting on an organizational level		
I am creating new recovery programs, representation at the health agency … (Clinician/professional)	2%	13

Being natural		
I stay natural with everyone. (User of mental health services)	2%	14

Paying attention to language		
I remove inadequate vocabulary: - user; services user. Person first! (Clinician/professional)	1%	15

**Table 2:** Themes and corresponding categories of strategies.

Theme	Categories of strategies
Education	Educating/teaching Giving successful examples Acting on an organizational level Paying attention to language

Contact	Sharing/encouraging disclosure Meeting/coming close to

Protestation	Defending rights Reframing words

Person centered	Normalizing Accepting/respecting Listening/caring

Working on recovery and social inclusion	Working on recovery Working on social inclusion

Reflexive consciousness	Doing introspective work Being natural
